# Targeting hypertension through natural phenolics: The multifaceted role of cinnamic acid and its derivatives

**DOI:** 10.1007/s00424-026-03163-2

**Published:** 2026-03-26

**Authors:** Samuel S. P. de Araujo, Brenda L. B. dos Santos, Waldilleny R. A. de Moura, Leonardo G. Rodrigues, Luiz G. S. Branco, Helio C. Salgado, Renato N. Soriano, Marina de T. Durand, João Paulo J. Sabino

**Affiliations:** 1 Multicenter Graduate Program in Physiological Sciences, Department of Biophysics and Physiology, Federal University of Piauí, Teresina, PI Brazil; 2 Graduate Program in Pharmaceutical Sciences, Department of Biophysics and Physiology, Federal University of Piaui, Teresina, PI Brazil; 3 Department of Basic and Oral Biology, Ribeirão Preto School of Dentistry, University of São Paulo, Ribeirão Preto, SP Brazil; 4 Department of Physiology, Medical School of Ribeirão Preto, University of São Paulo, Ribeirão Preto, SP Brazil; 5 Division of Physiology and Biophysics, Life Sciences Institute, Federal University of Juiz de Fora-campus GV, MG Governador Valadares, Brazil; 6 Medical School, University of Ribeirão Preto, Ribeirão Preto, SP Brazil; 7 Department of Biophysics and Physiology, Federal University of Piauí, University Avenue, Ininga, 64049- 550 Teresina Brazil

**Keywords:** Hypertension, Cinnamic acid, Phenolic acids, Renin-angiotensin system, Oxidative stress, Inflammation

## Abstract

Hypertension is a serious global public health problem that significantly increases the risk of cardiovascular diseases. Its etiology involves inflammatory processes, oxidative stress, and imbalances in blood pressure (BP) regulatory systems. In this context, the search for new therapeutic agents remains a major challenge, highlighting natural compounds such as cinnamic acid (CA) and its derivatives, including caffeic acid (CAF) and ferulic acid (FA). This review aimed to investigate the antihypertensive, anti-inflammatory, and antioxidant potential of CA and its derivatives. The phenolic acids analyzed, particularly CA and FA, exhibited greater inhibitory activity on angiotensin-converting enzyme (ACE), whereas CAF showed lower activity. Furthermore, cinnamic acid and its derivatives increased nitric oxide (NO) production, contributing to vasodilation. Regarding the inflammatory response, studies on phenolic acids demonstrated a reduction in the pro-inflammatory cytokines IL-1β, IL-6, IL-8, and TNF-α, accompanied by an increase in the anti-inflammatory cytokine IL-10, indicating a relevant anti-inflammatory effect. Similarly, in the antioxidant system, increased levels of superoxide dismutase (SOD) and glutathione (GSH) were observed, along with a reduction in the pro-oxidant marker malondialdehyde (MDA). Additionally, the DPPH and ABTS assays demonstrated greater free radical scavenging activity for CA, CAF, and FA derivatives. Taken together, these findings suggest that phenolic acids are promising candidates for the treatment of hypertension due to their favorable safety profile, with CA standing out as the main compound with hypotensive effects.

**Graphical Abstract:**

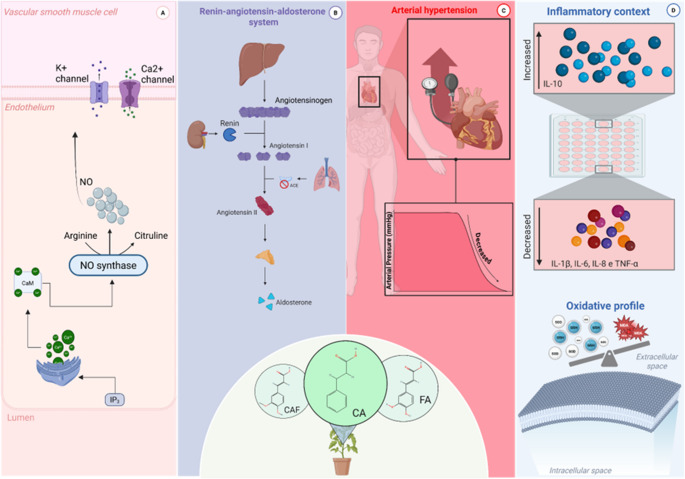

## Introduction

In this century, cardiovascular diseases have haunted the world due to the high rate of related morbidity and mortality [1]. Consequently, arterial hypertension (AH) has gained prominence due to its multifaceted and nonspecific development, worsening this alarming scenario [2]. AH has become a major challenge, as despite the existence of different pharmacotherapeutic approaches, their frequent and prolonged use culminates in resistance to traditional medications, side effects, and poor treatment adherence [3].

**Fig. 1:**
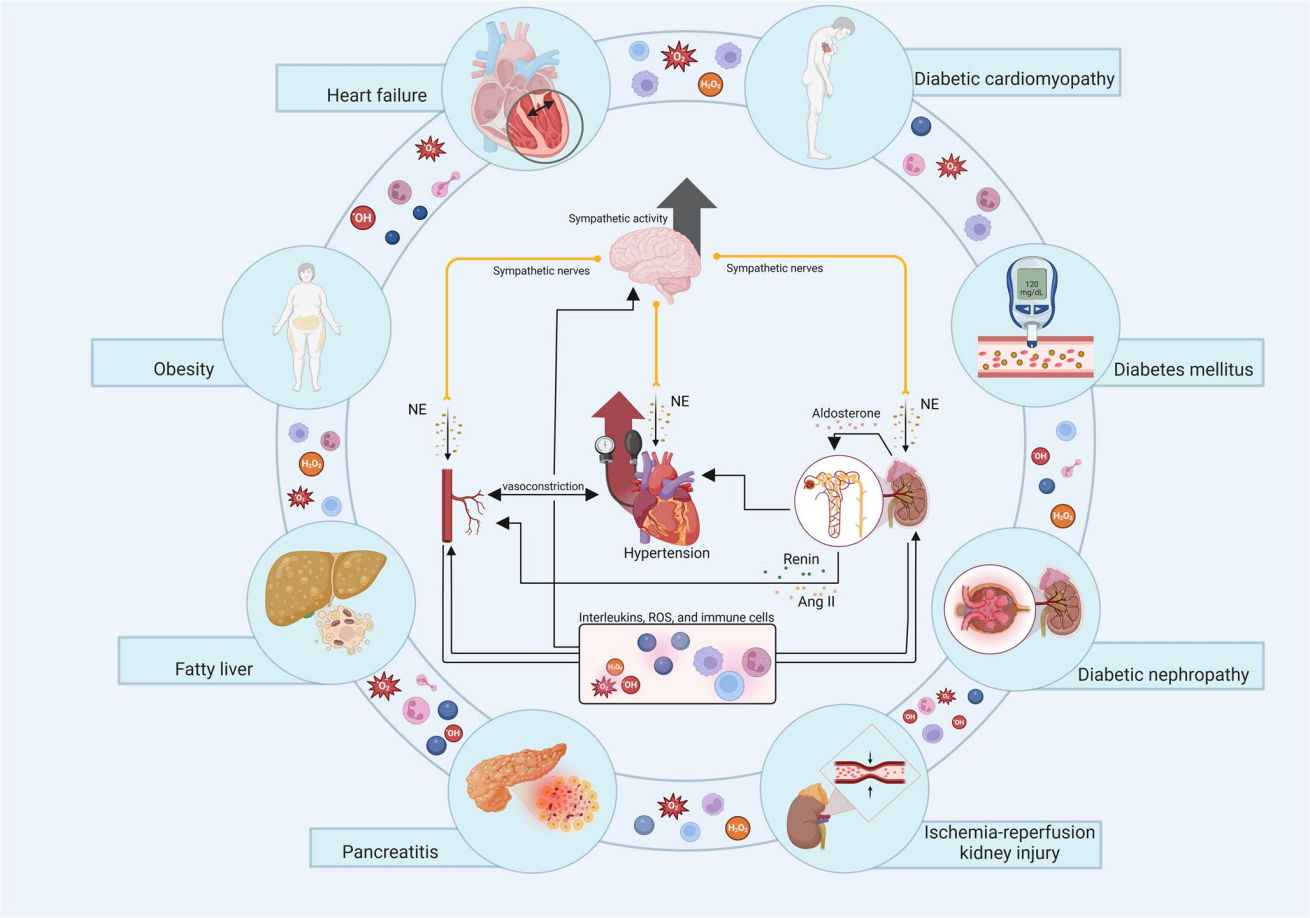
Mechanisms involved in hypertension. Exacerbated inflammation and oxidative stress lead to autonomic imbalance and sympathetic hyperactivity, increasing norepinephrine release. These processes amplify tissue damage and contribute to cardiovascular and metabolic diseases such as heart failure, diabetic cardiomyopathy, diabetes mellitus, nephropathy, and obesity. Angiotensin II (Ang II); norepinephrine (NE); reactive oxygen species (ROS). Figure created using BioRender.com

Furthermore, the inflammatory process and oxidative stress (OS) contribute to the maintenance and worsening of hypertension, given the close interrelationship between the two, which also contributes to sympathetic hyperactivity [4–6]. Inflammation is characterized by the recruitment of immune and non-immune cells and is a physiological condition developed throughout evolution to promote tissue repair and protection against infectious agents [7]. OS is described as an imbalance between the production and elimination of reactive oxygen species (ROS) [8]. At high concentrations, ROS impair vascular tissue homeostasis, causing damage to DNA and proteins/enzymes and promoting lipid peroxidation [9]. This condition intensifies inflammatory signaling and, consequently, contributes to the development and maintenance of hypertension [10]. Furthermore, an uncontrolled inflammatory and pro-oxidant state may be present in other clinical conditions, such as heart failure, diabetic cardiomyopathy, and renal ischemia [11–13], as well as metabolic conditions (diabetes, obesity, diabetic nephropathy, fatty liver disease, among others) [13–16]. Figure 1 summarizes these aspects.

In this context, natural products with anti-inflammatory, antioxidant, and/or antihypertensive properties emerge as promising candidates for combating these diseases [17]. Among these, phenolic compounds stand out, such as cinnamic acid (CA) and its derivatives (including trans-cinnamic, caffeic, and ferulic acids), which are natural bioactives with therapeutic potential for the aforementioned conditions (Tables 1 and 2) and present a lower risk of adverse effects compared with conventional therapies [18–20].

**Table 1:** Results from in vivo and in vitro studies on the antihypertensive effects of cinnamic acid and its derivatives

Compound	Animal species and experimental model	Dose/ duration	Results in hypertension	Reference
Cinnamic acid	In vivo - Obese / diabetic mice	25–50 mg/kg/ day (P.O for 4 weeks)	↑ Nitric oxide (NO) production via eNOS ↓ Vascular stiffness ↑ Endothelium-dependent vasodilation	(Bai et al. 2025)
	In vivo - Hypertensive female rats	Major comp.onent of the extract 10 mg/kg/ day (P.O for 10 weeks)	↓ Blood pressure ↑ Vascular lumen ↓ Kidney damage	(Trejo -Moreno et al. 2018)
	Ex vivo - Rats on a high- salt diet (aortic rings, isolated heart, and right atrium) In vivo - Rats on a high- salt diet	10 µg/mL 1, 3, 10, 30, 100 µg/kg	↑ Vasorelaxation ↑ Suppression of cardiac contraction force ↑ HR ↑ Suppression of coronary blood flow ↓ Right atrial contraction force and rate ↓ Blood pressure (dose- dependent) ↓ MAP ↓ SBP ↓ DBP	(Shah et al. 2024)
	In vivo - SHR rats In vitro - standard assays in controlled media	Amaranthus cruel leaf extract containing 1.2–2.8 mg/g cinnamic acid in 1 g of dry extract 200 mg/kg/ day (p.o 4–6 weeks) 40 µL of 1 mg/ mL sample extracts	↑ Vasorelaxation ↑ ACE inhibition	(Araujo -León et al. 2024)
	In vitro – primary neonatal rat cardiomyocytes and H9c2 cells In vivo – Mice with Angiotensin II- induced hypertension	4 µM – 500 µM 60 and 300 mg/kg (p.o for 14 days)	↓ Cardiomyocyte hypertrophy and H9c2 ↓ Nppb expression ↓ Blood pressure ↓ Cardiac index ↓ Left ventricular hypertrophy ↓ Fibrotic changes of the left ventricle	(Cui et al. 2025)
*Cinnamic acid derivatives*	*Ex vivo -* aortic rings from SHR (hypertensive animals)	Hydroethanolic extract of *P. barbatus* leaves Extract constituents: syringic acid, coumaric acid, caffeic acid, p- coumaroyl malate, rosmarinic acid, and di -O-methyl caffeic acid 0.3-1,000 µg/mL	↑ Vasorelaxation Voltage-gated K^+^channels Ca^2+^ activated K^+^channels Transmembrane Ca ^2+^ channels	(Moser et al. 2023)
	*In vivo - Male* *Wistar* rats and spontaneously hypertensive rats (SHR)	Lyophilized gel reconstituted in serum *Lycopersicum esculentum L* Chlorogenic acid was most abundant in all samples, ranging from 29.5 (Week 4) to 90.7 mg/kg body weight (Week 2) in gel and from 26.1 (Week 4) to 92.3 mg/kg body weight (Week 2) in serum and caffeic acid from 8.11 (Week 4) to 9.85 mg/kg body weight (Week 2) in gel and from 3.82 (Week 4) to 3.36 mg/kg body weight (Week 2) in serum) 400 mg of lyophilized gel in 12 mL of saline (orally for 4 weeks)	↓ SBP in SHR ↓ HR in SHR ↓ Aortic thickness in SHR	(Marcolongo et al., 2020)
	*In vivo -* Healthy men and women aged 20 to 64 years	Adlay Tea *Coix lacryma-jobi* (Gallic acid 0.035 g/ mL, 4-hydroxybenzoic acid 0.089 g/ mL, vanillic acid 0.266 g/ mL, caffeic acid 0.502 g/ mL, syringic acid 0.348 g/ mL, p- coumaric acid 0.647 g/ mL, ferulic acid 0.070 g/ mL and ethyl ferulate 0.065 g/ mL) 500 mL (p.o for 8 weeks)	+ 40 years ↓ arterial pressure volume index ↓ arterial pulse velocity index	(Oya et al., 2024)
	*In vivo -* Spontaneously hypertensive rats (SHR)	Ethyl acetate fraction of *Citrullus colocynthis* (phenolic compound concentration of 289.4 mg/g of dry plant material, including sinapic, ferulic and caffeic acids) 250 and 500 mg/kg (p.o for 28 days)	↓ PAM ↓ PAS ↓ PAD No change in HR	(Iftikhar et al., 2023)
*Caffeic acid*	*Ex vivo -* Male BALB/c mice (aortic rings)	Extract obtained from frozen blackberries (FrozAr) (acid caffeic acid, 3752.98 µg/g) and dried blackberries (DryAr) (caffeic acid, 4224.30 µg/g) 100 µg/ mL	Presence of elevated Ang II, LPS and GLUC, both extracts partially restored vascular relaxation	(Buda et al., 2024)
	*In vivo - Male* *Wistar* rats and spontaneously hypertensive rats (SHR)	Kukoamine which is derived from catechol or caffeic acid Single dose of 5 or 10 mg kg -1 of body weight of kukoamine A (p.o)	It had no effect on SBP, DBP and MAP in *Wistar rats* and SHR treated with kukoamine A	(Butts et al., 2022)
	In vitro *-* myofibroblast-like cell line CCD-18Co	50 and 10 µM	Low ACE inhibitory activity	(Zielinska et al., 2021)
*Ferulic acid*	Diabetic cardiomyopathy in Sprague-Dawley rats induced by 10% fructose and an injection of streptozotocin (40 mg/kg, ip .)	Ferulic acid 150 and 300 mg/kg (p.o for 5 weeks)	↓ AChE ↓ ACE ↓serum CK-MB levels Improvement in cardiac histology	(Salau et al., 2023)
	High-fat, high-carbohydrate diet in Sprague-Dawley rats	Ferulic acid 30 or 60 mg/kg (p.o. for 6 weeks)	↓ PAS ↓ PAD ↓ PAM ↓ ACE ↓ suppression of type 1 receptor ↓ vascular adhesion molecule 1	(Senaphan et al., 2025)

Nitric Oxide (NO). Endothelial Nitric Oxide Synthase (eNOS). Mean Arterial Pressure (MAP). Systolic Blood Pressure (SBP). Diastolic Blood Pressure (DBP). Oral Route (p.o). Spontaneously Hypertensive Rats (SHR). Angiotensin Converting Enzyme (ACE). Natriuretic Peptide Type B (Nppb). Blood Pressure (BP). Angiotensin II (Ang II). Ion Potassium (K^+^). Calcium Ion (Ca^2+^). Heart Rate (HR). Lipopolysaccharide (LPS). High glucose (GLUC). Acetylcholinesterase (AChE). Creatine Kinase-MB (CK-MB)

**Table 2:** Results from in vivo and in vitro studies on the anti-inflammatory and antioxidant effects of cinnamic acid and its derivatives

Compound	Animal species and experimental model	Dose/ duration	Anti-inflammatory and antioxidant effects	Reference
*Cinnamic acid*	In vivo *-* Obese/diabetic mice	25–50 mg/kg/day (p.o for 4 weeks)	↓Oxidative stress in aorta ↑ PPARδ, Nrf2/HO-1, AMPK/Akt/eNOS	(Bai et al. 2025)
	In vivo - Hypertensive female rats	Major component of the extract. 10 mg/kg/day (p.o for 10 weeks)	↓ O^− 2^ ions ↓ Kidney inflammation	(Trejo-Moreno et al. 2018)
	In vitro - standard tests in controlled media	*Amaranthus* leaf extract *cruel* containing 1.2–2.8 mg/g of cinnamic acid in 1 g of dry extract mL sample extracts	↓ ROSs	(Araujo-León et al. 2024)
	In vitro *-* primary neonatal rat cardiomyocytes and H9c2 cells	4 µM – 500 µM	↓Nppb expression ↓Mitochondrial SOD ↓ STAT3 and ERK1/2	(Cui et al. 2025)
*Cinnamic acid derivatives*	*In vitro -* Human hepatocellular carcinoma cells (HepG2, ATCC HB-8065) and Rat cardiomyocytes (H9c2, ATCC CRL-1446)	Biologically active derivatives of CA (cinnamic acid): (R, S)-(E)-N-(1-hydroxy-3-methylbutan-2-yl) cinnamamide), (E)-3-(4-chlorophenyl)-N-(1-hydroxy-2-methylpropan-2-yl) acrylamide ) and (E)-1-(4-hydroxypiperidin-1-yl)-3-phenylprop-2-en-1-one) Concentration of 25 µM	↓ ROSs	(Koczurkiewicz-Adamczyk et al., 2021)
	*In vivo - Male* *Wistar* rats and spontaneously hypertensive rats (SHR)	Lyophilized gel reconstituted in serum *Lycopersicum esculentum L (* Chlorogenic acid most abundant in all samples, ranging from 29.5 (Week 4) to 90.7 mg/kg body weight (Week 2) in gel and from 26.1 (Week 4) to 92.3 mg/kg body weight (Week 2) in serum and Caffeic acid 8.11 (Week 4) to 9.85 mg/kg body weight (Week 2) in gel and from 3.82 (Week 4) to 3.36 mg/kg body weight (Week 2) in serum) 400 mg of lyophilized gel in 12 mL of saline (orally for 4 weeks)	↓ TNFα	(Marcolongo et al., 2020)
	*In vitro -* DPPH radical scavenging assay, ABTS scavenging assay and Determination of Reducing Power *In vivo -* Spontaneously hypertensive rats (SHR)	Ethyl acetate fraction of *Citrullus colocynthis* (phenolic compound concentration of 289.4 mg/g of dry plant material, including sinapic, ferulic and caffeic acids ) Fraction of *Citrullus colocynthis* 1–100 µg/mL - DPPH Fraction of *Citrullus Colocynthis* 10–10,000 µg/mL – ABTS Fraction of *Citrullus Colocynthis* 0.625–10.0 mg - Reducing power Ethyl acetate fraction of *Citrullus Colocynthis* 250 and 500 mg/kg	↑ DPPH radical scavenging activity ↑ ABTS radical scavenging activity ↑ Reductive activity ↑ SOD ↑ GSH ↑ NOx ↑ TAC ↓ MDA	(Iftikhar N., et al., 2023)
*Caffeic acid*	*Ex vivo -* Male BALB/c mice (aortic rings)	Extract obtained from frozen blackberries (FrozAr) (acid caffeic acid, 3752.98 µg/g) and dried blackberries (DryAr) (caffeic acid, 4224.30 µg/g) Evaluation of hydrogen peroxide production (1, 10, 50, 75, 100, 500 µg/ mL) Evaluation of the catalase-catalyzed effect of H2O2 (1, 5, 7.5, 10, 25, 50, 75, 100, 150, 500 µg/mL)	In the presence of elevated Ang II, LPS and GLUC, both extracts: ↓ ROS	(Buda et al., 2024)
	In vitro *-* myofibroblast-like cell line CCD-18Co	50 and 10 µM	↓ IL-1β ↓ PGE2 formation ↓ IL-8 ↑ Chelation activity	(Zielinska et al., 2021)
	*In vivo -* *Wistar* rats with intraperitoneal injection of streptozotocin (STZ)	10 and 50 mg/kg (p.o for 30 days)	↑ IL-10 gene expression ↓ MPO Reversed the increase in reactive species ↓ Lipid peroxidation ↓ Protein oxidation levels	(Castro et al., 2021)
	*In vivo -* Swiss mice with alloxan monohydrate-induced diabetes	50 mg kg/1 (ip daily for seven days)	↓ Erythrocyte hemolysis ↓ Lipid peroxidation ↓ MDA	(Oršolić et al., 2021)
*Ferulic acid*	*In vitro -* DPPH free radical scavenging and L6 myoblast cell testing *In vivo -* Sprague rats Dawley underwent renal laparotomy surgery to induce ischemia/ reperfusion kidney injury	*Ocimum* extracts *gratissimum* and *Musanga cecropoides*,* were respectively: 0.21% and 0.08%* chlorogenic acid, 0.2% and 0.12% caffeic acid, 0.01% and 47% apigenin or derivatives and in *Ocimum gratissimum contained 1.2%* chicoric acid Isolated ferulic acid − 100 mg/kg p.o, 24 h before ischemia Combined with FA − 100 mg/kg p.o, 24 h before ischemia and 5 mg/kg ip. of zinc oxide nanoparticles for 2 h before ischemia	↑ Cell protection against oxidative stress from H_2_O_2_ ↑ DPPH IC50 inhibition ↑ Antioxidant activity ↓ MDA ↑ CAT ↑ SOD ↑ HO-1 expression ↑ NRF2 expression ↓ TNF-α expression ↑ HIF-1α expression	(Awadalla et al., 2021)
	*In vivo -* Sprague rats Dawley with isoproterenol (ISO)-induced heart failure	25 and 50 mg/kg, for 4 days before administration of ISO and concomitantly	↑ SOD ↑ GSH-Px ↓ MDA	(Zhang et al., 2021)
	*In vivo -* Sprague-Dawley rats on a high-cholesterol, high-fat diet for the induction of nonalcoholic fatty liver disease *In vivo -* Sprague-Dawley rats fed a high-cholesterol, high-fat diet for the induction of nonalcoholic fatty liver disease	20 mg/kg/day (p.o for 4 weeks) 20 mg/kg/day (p.o for 4 weeks)	↑ SOD ↓ MDA ↓ MDA ↑ SOD ↓ MDA ↓ IL-1β ↓ IL-6 ↓ TNF-α	(Wei et al., 2021)
	*In vivo -* Diabetic cardiomyopathy in Sprague-Dawley rats, induced by 10% fructose and an injection of streptozotocin (40 mg/kg, ip .)	150 and 300 mg/kg (p.o for 5 weeks)	↑ GSH ↑ CAT ↑ SOD ↓ MDA	(Salau et al., 2022)
	*In vitro −* 3T3-L1 adipocytes	1, 10 and 50 µM for 24 h after LPS	↓ TNF‑α ↓ IL-6 ↓ IL-1β ↓ MCP-1	(Park; Han, 2024)
	*In vitro -* DPPH radical scavenging assay, ABTS scavenging assay *In vivo - Obese* Wistar rats fed a high-fat diet	C. sinensis (ranging from 0.25 to 5 mg/ mL) 7.15 mg/kg (p.o for 9 weeks)	↑ DPPH radical scavenging activity ↑ ABTS radical scavenging activity ↓ IL-1β ↓ IL-6 ↓ IL-8 ↑ IL-10 ↓ TNF-α ↓ CRP ↑ CAT ↑ GHS-Px ↑ SOD ↓ TBARS	(Gomes Schmitt et al., 2025)
	*In vivo -* Male C57BL/6J mice with diabetic nephropathy	200 mg/kg FA (p.o for 8 weeks)	↓ NLRP3 ↓ IL-1β	(Ma et al., 2022)

Oral Route (p.o). Peroxisome Proliferator-Activated Receptor delta (PPARδ). Nuclear Factor Erythroid 2-Related Factor 2 (Nrf2). Heme Oxygenase-1 (HO-1). AMP- Activated Protein Kinase (AMPK). Protein Kinase B (Akt). Oxygen Ion (O^2−^ ). Reactive Oxygen Species (ROS). Signal Transducer and Activator of Transcription 3 (STAT3). Extracellular signal-regulated protein kinase 1/2 (ERK1/2). Cinnamic acid (CA). Tumor Necrosis Factor alpha (TNFα). 2,2-diphenyl-1-picrylhydrazyl (DPPH). 2,2’-azinobis acid ( 3-ethylbenzothiazoline-6-sulfonic acid (ABTS). Superoxide Dismutase (SOD). Glutathione (GSH). Nitrogen Oxides (NOx). Total Antioxidant Capacity (TAC). Malondialdehyde (MDA). Angiotensin II (Ang II). Lipopolysaccharide (LPS). High glucose (GLUC). Interleukin 1 beta (IL-1β). Interleukin 8 (IL-8). Interleukin 10 (IL-10). Myeloperoxidase (MPO). Hydrogen Peroxide (H_2_O_2_). Glutathione Peroxidase (GSH- Px ). Interleukin 6 (IL-6). Intraperitoneal ( ip ). Catalase (CAT). Monocyte chemotactic protein-1 (MCP-1). C- reactive protein (CRP). Thiobarbituric Acid Reactive Substances (TBARS). NOD-like receptor family pyrin domain-containing 3 (NLRP3)

Therefore, this review aims to elucidate the bioactivity and potential mechanisms of action of CA and its derivatives in the context of experimental hypertension, with particular emphasis on associated alterations such as inflammation and oxidative stress.

## Characteristics of CA and its derivatives

**Fig. 2:**
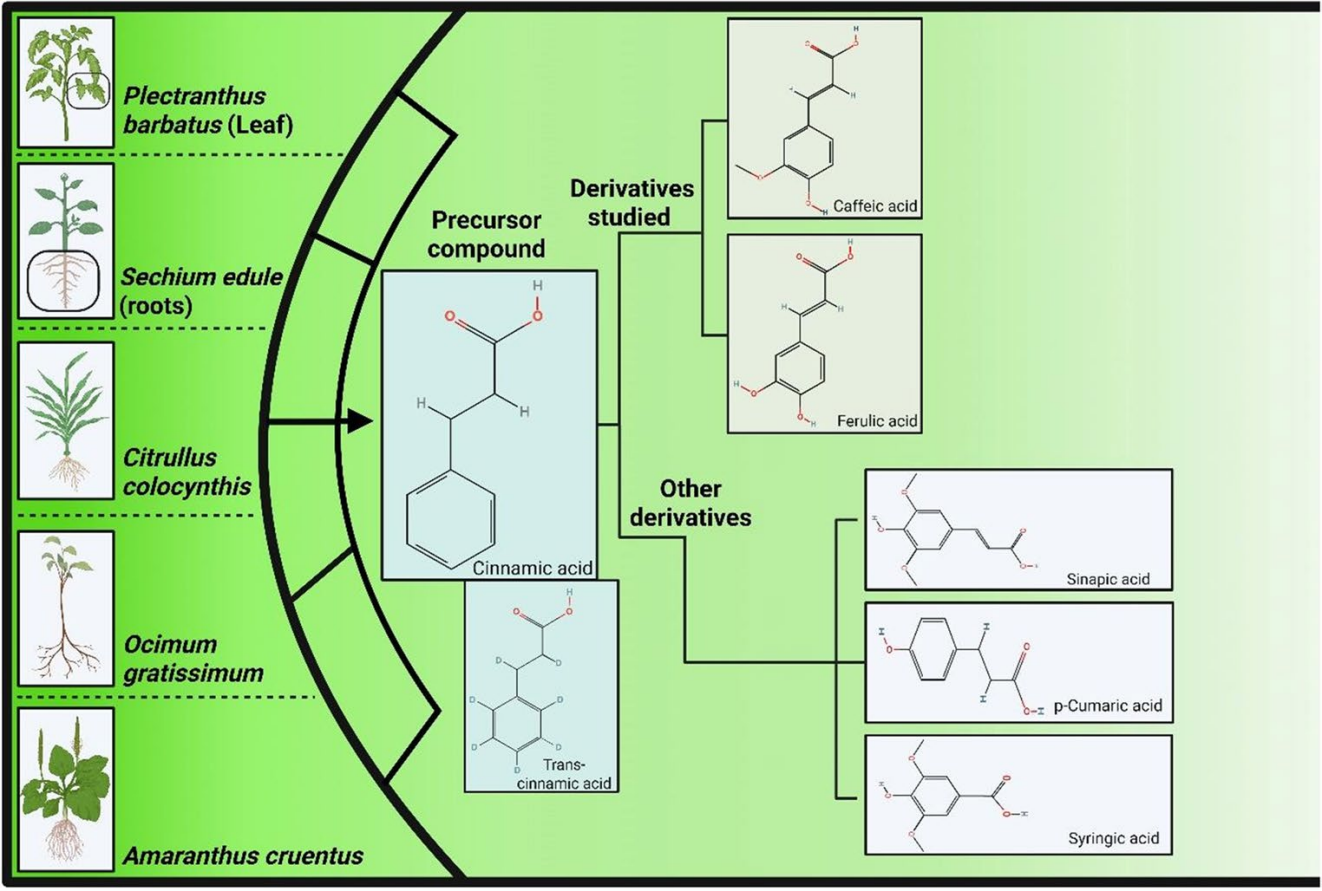
Distribution of cinnamic acid and related phenolic compounds in plant species. Plant species containing cinnamic acid, its derivatives, and its precursors, caffeic and ferulic acids. These species exhibit extracts in which cinnamic acid or its precursors (caffeic or ferulic acids) are major components, along with important derivatives such as sinapic, p-coumaric, and syringic acids. Figure created using BioRender.com

Polyphenols are among the most important and abundant phytochemicals in human diets. Phenolic acids are generally classified into two main groups: benzoic acids, which contain seven carbon atoms (C6–C1), and CA, which contains nine carbon atoms (C6–C3) [21]. This review analyzed the cis and trans forms of CA, caffeic acid (CAF), and ferulic acid (FA), which are widely present in plants (vegetables and fruits). However, sinapic, coumaric, and chlorogenic acids are also found in extracts of CA derivatives (Fig. 2).

In recent years, CA and its derivatives have been investigated and explored due to their remarkable biological properties, making them an attractive platform for various cutting-edge applications, ranging from food packaging to electrochemistry and biomedicine [22]. Due to their widespread occurrence in plants, low toxicity, and high biological activity, CA derivatives are considered food additives (antioxidants, preservatives) or pharmacologically active compounds [23].

CA is a monocarboxylic acid consisting of acrylic acid with a phenyl substituent at the 3-position. It is named after cinnamon (Cinnamomum zeylanicum bark) but can also be found in Panax ginseng, fruits, whole grains, vegetables, and honey [24–26]. As previously described, CA occurs naturally in either the cis or trans form, although the trans form is predominant due to its greater stability compared to the cis form [27, 28].

CAF is one of the most common phenolic acids found in plants and vegetables [29]. Natural sources rich in CAF include coffee beans, and the content depends on the roast and preparation method [30]. In addition to coffee, fruits such as apples, pears, cherries, and grapes also contain significant amounts of this phenolic compound [29]. CAF is present in the seeds of plants belonging to the Camellia genus, as well as in thyme and mint [24], sweet potato (Ipomoea batatas L.), and artichoke (Cynara cardunculus L.) [21]. Its presence has also been demonstrated in some mushrooms, including Agaricus bisporus, Coprinus atramentarius, Morchella elata, and Laetiporus sulphureus [31]. Like other phenolic compounds, CAF has also been studied for its antioxidant [32, 33] and anti-inflammatory properties [34, 35]. FA occurs in significant quantities in wheat bran, tomatoes, cucurbits, thyme, sage, marjoram, and rosemary [36]. This compound exhibits anti-inflammatory [37, 38] and antioxidant properties [3–40].

### Cinnamic acid (CA)

#### Antihypertensive and vasorelaxant effects of CA

A literature search revealed studies describing relevant therapeutic properties of CA, including consistent effects in attenuating hypertension and the underlying mechanisms leading to its development in different experimental models (Table 1). In vivo, CA exhibited antihypertensive and vascular protective effects. In female hypertensive mice (induced by injection of angiotensin II (Ang II) 0.1 µg/kg/day for 10 weeks), CA-rich *Sechium edule* root extract (10 mg/kg/day, p.o.) controlled BP and increased vascular lumen [41]. Similarly, other extracts induced positive results in cardiovascular reactivity, as Araujo-León et al. [42] evaluated leaf and inflorescence extracts of red amaranth (*Amaranthus cruentus*) containing CA. The findings showed that the extracts promoted vasorelaxation in aortic rings; moreover, they also demonstrated possible antihypertensive action, as an in vitro approach showed that these compounds were effective in inhibiting angiotensin-converting enzyme (ACE) [42]. Although the two studies cited above present promising results, they should be analyzed carefully, since CA was obtained through an extract and not in isolation. Therefore, it cannot be ruled out that some of the beneficial effects were promoted by other compounds present in the extract.

On the other hand, some studies suggest the therapeutic potential of isolated CA, as CA administration (for 4 weeks, 40 mg/kg/day, gavage) to diet-induced obese mice significantly reduced BP, and, ex vivo, CA (1–30 µM) significantly improved endothelium-dependent relaxation in mouse aortas. This effect was associated with activation of the peroxisome proliferator-activated receptor (PPAR), nuclear factor erythroid 2 (NRF2)/heme oxygenase-1 (HO-1), and AMP-activated protein kinase (AMPK)/protein kinase B (Akt)/endothelial nitric oxide synthase (eNOS) signaling pathways, which contributed in part to its protective action against endothelial dysfunction and hypotensive effect [43].

Furthermore, reports in the literature indicate that the beneficial effects of isolated CA are not limited to metabolic diseases. In experimental models of hypertension, CA has also been shown to promote positive effects on cardiovascular parameters through various signaling pathways. Intravenous injection of CA caused a dose-dependent decrease (1–30 µg/kg) in systolic (SBP) and diastolic blood pressure (DBP) in hypertensive rats (induced by a high-salt diet) and a vasorelaxant effect in isolated aortic rings through the release of substances such as nitric oxide (NO) and prostaglandin I2 (PGI2), mediated by muscarinic receptors [44]. In another model of hypertension (induced by Ang II subcutaneous administration for 14 days), CA administered orally (60–300 mg/kg) reduced BP, left ventricular hypertrophy, and cardiac fibrosis [45]. The deleterious cardiac effects induced by Ang II occur, in part, through increased STAT3 activity and mitochondrial impairment. It was observed that cardiomyocytes treated with CA (100 µM) showed reduced pSTAT3 levels. Furthermore, although ERK1/2 activation contributes to Ang II-induced hypertrophy, CA treatment reduced pERK1/2 levels in these cells [45].

Therefore, a substantial body of evidence supports the hypotensive effect of CA, as well as its possible mechanisms of action in experimental models of hypertension and diabetes. However, the findings cannot yet be considered conclusive, as further analyses are still required, including: (a) evaluation of the safety and toxicity profile; (b) limited evidence in female subjects; (c) comparative analyses with established antihypertensive medications; and (d) the lack of clinical data. Thus, comprehensive preclinical and clinical studies are necessary to confirm the therapeutic potential of CA.

### Antioxidant and anti-inflammatory effects of CA

As previously reported, there is a growing body of evidence suggesting that the development and maintenance of hypertension are also due to oxidative stress and, consequently, inflammation [46]. Therefore, it is expected that new therapeutic approaches for patients unresponsive to existing medications will prioritize the development of antihypertensive drugs that also act to reduce oxidative stress and inflammation. In this context, CA has demonstrated robust antioxidant capacity (Table 2). In obese/diabetic mice (induced by a high-fat diet with 60% kcal from fat for 12 weeks), CA (40 mg/kg/day), administered during the final 4 weeks of the high-fat diet, promoted the activation of signaling pathways involving peroxisome proliferator-activated receptor delta (PPARδ), NRF2/HO-1, and AMPK/Akt/eNOS, in addition to decreasing OS. This contributed to improving endothelial dysfunction in the aorta of these animals [43]. In female mice with hypertension (induced by Ang II, i.p., 0.1 µg/kg/day for 10 weeks), a CA-rich extract attenuated OS by decreasing superoxide ion (O₂⁻) levels [41].

In another study, a pharmacological screening of aqueous methanol extracts from *Amaranthus cruentus* leaves and inflorescences was performed to determine their 2,2-diphenyl-1-picrylhydrazyl (DPPH) radical scavenging capacity. In the DPPH assay, extracts from *Amaranthus cruentus* leaves and inflorescences were observed to neutralize the DPPH radical by 52% and 46%, respectively. Notably, the level of antioxidant efficacy observed in both treatments was considered promising, as a similar antioxidant capacity was observed in the positive control, ascorbic acid [42].

The antioxidant role of CA was also investigated in H9c2 cardiomyocytes treated with Ang II, in which the induction of cardiac hypertrophy and impairment of mitochondrial membrane potential (MMP) after Ang II exposure were observed [45]. Conversely, improvement in cardiac hypertrophy and MMP was observed in Ang II-exposed cells treated with 100 µM CA. Moreover, MitoSOX Red superoxide staining revealed that mitochondrial superoxide levels significantly decreased in CA-treated cells, as demonstrated by mitochondrial functional assays, indicating that the antihypertrophic action of CA involves attenuation of Ang II-triggered mitochondrial impairment in cardiomyocytes [45].

In the study by Cui et al. [45], exposure to IL-6, which canonically activates STAT3 and ERK1/2 and triggers prohypertrophic responses, was subsequently applied to cardiomyocytes. As expected, distinct STAT3 activation was readily observed after this exposure. However, CA dose-dependently attenuated IL-6-induced STAT3 activation in cardiomyocytes. Significant reductions in pSTAT3 levels were observed in cells treated with 100 and 500 µM CA. Similarly, the IL-6-stimulated elevation of pERK1/2 levels was also dose-dependently attenuated by CA. Significantly lower pERK1/2 levels were observed in cells treated with 20, 100, and 500 µM CA, but not in those treated with 4 µM CA.

Interestingly, a study focused on analyzing OS in the pancreas also confirmed the antioxidant capacity of CA. In this regard, in acute pancreatitis (induced by L-arginine injections, 250 mg/100 g, i.p., for 10 days, followed by gamma irradiation), it was observed that after treatment with CA nanoparticles (60 mg/kg/day, p.o., for 21 days), male albino rats exhibited an improved redox profile, given the decrease in OS biomarkers such as malondialdehyde (MDA) and oxidized glutathione (GSSG), along with an increase in reduced glutathione (GSH) and a decrease in the GSSG/GSH ratio [46]. Furthermore, in this same study, it was found that the mitogen-activated protein kinase (MAPK) family signaling pathway (p38, ERK1/2, JNK), the expression of nuclear factor kappa B (NF-κB), NLR family pyrin domain containing 3 (NLRP3), and apoptotic markers such as caspase-3 and apoptosis signal-regulating kinase 1 (ASK1) were decreased, indicating the important role of CA in cellular regulation aimed at attenuating OS [47].

Therefore, the results regarding OS and inflammatory parameters (Table 2) indicate that the antihypertensive and vasorelaxant effects of CA involve the attenuation of signaling pathways present in states of oxidative stress and inflammation.

### Cinnamic acid (CA) derivatives

#### Antihypertensive and vasorelaxant effects of CA derivatives

Another set of data also suggests that not only does CA possess promising antihypertensive properties, but its derivatives do as well (Table 1), indicating that this class of natural products is a potential candidate for the treatment of hypertension. Among the studies on CA derivatives, the study by Moser et al. [48] showed that the hydroethanolic extract of *Plectranthus barbatus* leaves, rich in CA derivatives, flavonoids, diterpenoids, and organic acids (citric acid, syringic acid, coumaric acid, salvianic acid, CAF, p-coumaroyl malate, rosmarinic acid, and di-O-methyl caffeic acid), promoted vascular relaxation in aortic rings with functional endothelium, both from Wistar rats and spontaneously hypertensive rats (SHR). The relaxing effect occurred in a concentration-dependent manner (0.3–1000 µg/mL). Regarding the mechanisms involved in this effect, the participation of voltage-gated and Ca²⁺-activated K⁺ channels was observed, in addition to the involvement of transmembrane channels for Ca²⁺, suggesting that these ionic pathways contribute to the observed vasodilatory response [48].

There is also evidence of CA derivatives affecting other cardiovascular parameters in SHR. The locular gel/serum of *Lycopersicum esculentum* demonstrated excellent bioactivity through chlorogenic acid, which is derived from CA, more specifically from CAF, which was also evaluated and present in smaller quantities [49]. The lyophilized gel, reconstituted in serum, consisted of 400 mg of lyophilized gel in 12 mL of serum and was administered orally for 4 weeks. After this period, it was observed that the gel was able to significantly reduce SBP and heart rate (HR) in SHR, in addition to having a vascular protective effect, reducing aortic wall thickness [49].

The only human study on CA derivatives evaluated the health benefits of Adlay tea intake (500 mL for 8 weeks) on immune homeostasis and vascular endothelial functions in men and women aged 20 to 64 years [50]. Analysis of phenolic components detected several polyphenols in Adlay tea, including gallic acid (0.035 g/mL), 4-hydroxybenzoic acid (0.089 g/mL), vanillic acid (0.266 g/mL), CAF (0.502 g/mL), syringic acid (0.348 g/mL), p-coumaric acid (0.647 g/mL), FA (0.070 g/mL), and ethyl ferulate (0.065 g/mL). The main result correlated with hypertension: in individuals over 40 years of age, the arterial pulse velocity index increased significantly only in the placebo group [50].

## Anti-inflammatory and antioxidant effects of CA derivatives

To evaluate the anti-inflammatory activity of the locular gel/serum of *Lycopersicum esculentum*, Marcolongo et al. [49] collected blood samples from SHR and Wistar Kyoto rats to determine serum levels of tumor necrosis factor alpha (TNF-α). As a result, after treatment (administered for 4 weeks), it was observed that only the gel/serum extract was effective in reducing this marker, performing better than treatment with captopril, which was used as a positive control.

Subsequently, the study by Iftikhar et al. [51] aimed to describe the effects of polyphenol-rich fractions from *Citrullus colocynthis* on their possible antioxidant and antihypertensive activities. Regarding the analyzed fractions, the ethyl acetate fraction stood out for presenting the highest concentration of phenolic compounds (289.4 mg/g of dry plant material), among which sinapic, ferulic, and caffeic acids were present. In vitro, this fraction demonstrated greater scavenging activity of DPPH radicals (SC50 6.2 µg/mL, scavenging concentration 50%) and 2,2′-azinobis(3-ethylbenzothiazoline-6-sulfonic acid) (ABTS) radicals (IC50 76.5 µg/mL). In ex vivo assays, the antioxidant capacity of the ethyl acetate fraction was tested in the blood plasma of SHR rats (treated with doses of 250 and 500 mg/kg for 28 days, p.o.). It was found that there was an improvement in the oxidative profile, given the increase in NOx, superoxide dismutase (SOD), GSH, and total antioxidant capacity (TAC), in addition to the reduction in MDA; the group that received the 500 mg/kg dose showed the most pronounced effects [51].

The biologically active derivatives of CA—(R, S)-(E)-N-(1-hydroxy-3-methylbutan-2-yl)cinnamamide, (E)-3-(4-chlorophenyl)-N-(1-hydroxy-2-methylpropan-2-yl)acrylamide, and (E)-1-(4-hydroxypiperidin-1-yl)-3-phenylprop-2-en-1-one—were tested (25 µM) in cell models of human hepatocellular carcinoma (HepG2, ATCC HB-8065) and rat cardiomyocytes (H9c2, ATCC CRL-1446). These cells were pre-incubated with the derivatives for the ROS-Glo H₂O₂ test, which measures hydrogen peroxide (H₂O₂) levels to evaluate ROS [52]. As a result, CA derivatives showed protective activity against doxorubicin-induced oxidative stress in the tested cell lines [52].

Therefore, CA derivatives have demonstrated significant therapeutic potential in experimental models and in vitro assays (Table 2), but their transposition to hypertension in humans remains a challenge. Since they are a mixture of compounds, it is difficult to attribute the beneficial effects to any specific compound. Therefore, more studies with isolated CA derivatives should be encouraged to facilitate result analysis and the possibility of proposing new therapies. Among the CA derivatives, the current review analyzed the main results of CAF and FA.

### Caffeic Acid (CAF)

#### Antihypertensive and vasorelaxant effects of CAF

In the study conducted by Buda et al. [53], the chemical composition of extracts obtained from blackberries through different processing methods was evaluated. The extract obtained from frozen fruits (FrozAr) showed a high content of hydroxycinnamic acids, with CAF standing out (3752.98 µg/g), as well as flavonols and anthocyanins. On the other hand, the extract obtained from dried fruits (DryAr) revealed a higher concentration of flavonols and hydroxycinnamic acids, including CAF (4224.30 µg/g) and chlorogenic acid (3024.26 µg/g). In functional assays performed on aortic rings isolated from BALB/c mice, previously exposed to Ang II, lipopolysaccharide (LPS), or high glucose concentrations (conditions that compromise the endothelial vasodilatory response to acetylcholine), it was observed that incubation with both extracts (100 µg/mL), DryAr and FrozAr, was able to partially restore vascular relaxation [53]. The fact that these studies used only extracts containing CAF makes the results less robust, making it impossible to state that the vasodilatory responses occurred exclusively as a result of CAF.

Furthermore, a study with kukoamines, which are organic compounds defined as derivatives of CAF and polyamines [54], showed no positive effect on cardiovascular parameters. In this study, kukoamine was used in its synthetic form, as kukoamine A, in a daily oral dose (5 or 10 mg/kg body weight) in SHR to evaluate its effect on SBP. As a result, it was observed that kukoamine A intake for 5 weeks did not attenuate SBP in the model evaluated [54]. These data do not corroborate studies with extracts containing CAF cited in the previous paragraph, indicating the need for more studies with isolated compounds. Furthermore, the digestion and absorption process may have limited the action of kukoamine, so studies with other doses of kukoamine should be encouraged.

Zielinska et al. [35] evaluated the effect of CAF (10 and 50 µM) on colon fibroblasts, subepithelial cells involved in modulating the ACE-induced inflammatory response. The results demonstrated low inhibition of ACE by CAF, indicating that although this signaling pathway is relevant, the compound showed a limited effect on its modulation. Therefore, based on the present experimental conditions, we can suggest that CAF exerts a diminished inhibitory effect on ACE, which is part of a well-described inflammatory signaling pathway.

As aforementioned, ACE and its pathological pro-inflammatory pathway, the ACE/Ang II/Ang II receptor type 1 axis, are involved in the progression of many diseases, playing an important role in the body’s regulation and inflammatory processes [55]. Overexpression of ACE and elevated levels of Ang II are directly linked to endothelial dysfunction, increased renal sodium reabsorption, and activation of inflammatory pathways, contributing to peripheral vascular resistance. Conversely, compounds that inhibit ACE have a beneficial effect on the vascular wall by inhibiting pathological wall remodeling [56].

Although CAF exhibits important properties that could mediate beneficial responses in hypertension, studies using both in vitro and in vivo models have shown only a partial vasorelaxant response. Furthermore, the scarcity of experiments with isolated CAF in hypertension models, or even in humans, limits the understanding of its real effects regarding the vasorelaxant and hypotensive response of the compound.

### Anti-inflammatory and antioxidant effects of CAF

In an ex vivo study, Buda et al. [53] evaluated the activity of two natural extracts from *Aronia melanocarpa*, namely DryAr and FrozAr, at the following concentrations: 1, 10, 50, 75, 100, and 500 µg/mL. The extracts were subjected to tests to evaluate their potential bioactivity on oxidative stress (OS) in young male mice (BALB/c strain, 4 weeks old, 25 g). The antioxidant activity of the extracts was assessed by inhibiting the production of H₂O₂ using spectrophotometry (FOX assay). For this purpose, aortic rings were incubated with OS-inducing agents: Ang II (100 nM), lipopolysaccharide [LPS (1 pM)], or high glucose (22.2 mM) [53].

The results of the FOX assay indicated that, in both control samples and in cells exposed to DryAr or FrozAr extracts, no significant changes in H₂O₂ levels were observed (medium only, without Ang II). In contrast, pre-incubation with Ang II alone promoted a substantial increase in H₂O₂ production; however, co-incubation with DryAr or FrozAr and Ang II resulted in a significant reduction in H₂O₂ levels at concentrations of 100 and 500 µg/mL [53].

Furthermore, the second FOX assay, with pre-incubation with LPS, showed results similar to those previously described [52]. However, in this assay, concentrations of 50, 100, and 500 µg/mL of both extracts were effective in reducing H₂O₂ [53]. The third FOX assay, with high glucose pre-incubation, also showed no significant changes in the control groups (DryAr or FrozAr alone); however, when the samples with high glucose concentration were exposed to DryAr, a reduction in H₂O₂ was observed at concentrations of 100 and 500 µg/mL. FrozAr (50, 100, 500 µg/mL) was able to reduce H₂O₂ in samples pre-exposed to high glucose, indicating greater versatility of FrozAr [53].

Thus, analyzing the OS results induced by Ang II, LPS, and high glucose, we can infer that both extracts were able to reduce H₂O₂ levels. However, the experimental approaches do not allow us to conclude which mechanism of action is responsible for the reduction of OS after treatment with the extracts.

In the same study [53], to more robustly describe the H₂O₂ inhibition capacity (%) of both extracts, concentrations of 1, 5, 7.5, 10, 25, 50, 75, 100, 150, and 500 µg/mL were subjected to tests to evaluate their catalase-like scavenging activity. For this purpose, an H₂O₂ solution (100 µM) was used as a negative control, while incubation with H₂O₂ plus catalase (100 U/mL) was used as a positive control. The findings show a difference between the control groups, with the classic result of catalase inhibiting H₂O₂ (%); however, DryAr and FrozAr extracts (100, 150, 500 µg/mL) were capable of inducing H₂O₂ inhibition (%) [53]. Thus, the authors suggested that these results partially explain the antioxidant capacity of the extracts.

As previously reported in other studies, some natural compounds containing CAF can exhibit physiological effects in various assays. With this in mind, Oršolić et al. [57] aimed to evaluate whether CAF (50 mg/kg/day for 7 days) affected lipid peroxidation in the liver, brain, and kidney under the pathophysiological condition of type I diabetes. Type I diabetes was induced in Swiss albino mice using alloxan. As a result, it was reported that the group of diabetic mice exhibited elevated levels of MDA. However, when treated with CAF, reduced MDA levels were found in liver and kidney samples. Furthermore, in the brains of both healthy and diabetic mice, attenuation of MDA levels was observed when treated with CAF.

Still evaluating the damage induced by OS, the osmotic fragility of erythrocytes and erythrocyte hemolysis were assessed. Regarding osmotic fragility (when exposed to 50% NaCl, which causes cell membrane rupture), CAF treatment protected erythrocytes. Regarding erythrocyte hemolysis (when exposed to H₂O₂), CAF reduced OS in the diabetic group.

Still in the search for new natural products with antidiabetic properties, Awwad et al. [58] investigated, in vitro, two plant bioactives: *Ocimum gratissimum* leaf extract and *Musanga cecropoides* stem bark extract, for their antioxidant capacity by scavenging DPPH free radicals and evaluating the protective effect of L6 cells against H₂O₂. The percentages of caffeic derivatives in the *Ocimum gratissimum* and *Musanga cecropoides* extracts were, respectively: 0.21% and 0.08% chlorogenic acid, 0.2% and 0.12% CAF, and 0.01% and 47% apigenin or derivatives; *Ocimum gratissimum* contained 1.2% chicoric acid. As a result of the DPPH assay, when compared to the standard control quercetin, both extracts were capable of inhibiting the evaluated free radicals; however, the *Musanga cecropoides* extract promoted a more pronounced response.

Subsequently, L6 myoblast cells were exposed to the different extracts at a final concentration of 50 µg/mL. When the cells were exposed to H₂O₂ (20 µM), pre-exposure to *Ocimum gratissimum* generated a protective effect against H₂O₂ (-21% mortality) compared to the untreated control (+ 42% mortality). Nonetheless, *Musanga cecropoides* caused a marked increase in L6 cell mortality (+ 22.5% mortality) [58].

In addition, Castro et al. [59] investigated the effects of CAF on OS and inflammatory processes in streptozotocin-induced diabetic Wistar rats. After treatment with CAF (10 and 50 mg/kg p.o., for 30 days), OS markers such as myeloperoxidase (MPO), ROS levels, thiobarbituric acid reactive species (TBARS), protein carbonylation, and thiol levels (total thiol [T-SH] and non-protein thiol [NPSH]) were quantified in blood plasma. CAF, at both concentrations studied, was able to decrease MPO, ROS, MDA, and protein carbonylation, while simultaneously increasing T-SH and NPSH levels. These results demonstrate CAF’s ability to attenuate OS caused by diabetes. However, it is worth noting that the results were not dose-dependent.

In the same study by Castro et al. [59], CAF was tested for its potential anti-inflammatory activity. For this analysis, inflammatory cytokines (IL-1β, IL-6, and IL-10) and acetylcholine-degrading enzymes, such as acetylcholinesterase and butyrylcholinesterase (AChE and BuChE, respectively), were evaluated. Based on the gene expression of inflammatory cytokines, it was observed that CAF at concentrations of 10 and 50 mg/kg was unable to attenuate the increase in IL-1β and IL-6. Conversely, IL-10, which has anti-inflammatory activity, was increased in both diabetic and healthy animals treated with CAF. Therefore, in the experimental model of streptozotocin-induced diabetes, CAF was able to reduce OS parameters but did not affect inflammatory cytokines, indicating that the effect of CAF under this experimental condition (dose and duration of treatment) may be limited.

Moreover, CAF, at non-toxic concentrations, reduced the effect of IL-1β (1 ng/mL) on PGE2 formation in colon myofibroblasts [35]. That is, the increase in PGE2 observed in myofibroblasts treated with IL-1β was attenuated in the presence of CAF at 10 and 50 µM. Beyond that, only a small reduction in IL-8 concentration was observed in the presence of CAF at the same concentrations, and DPPH values were only 25% lower than the radical scavenging activity of uric acid. In contrast, CAF showed very high chelating activity when compared to ascorbic acid and uric acid, which were used as positive controls [35]. Thus, the aforementioned results indicate the need for further studies to elucidate the potential beneficial effects of CAF on OS and inflammatory processes in colon myofibroblasts.**Ferulic acid (FA)**.

### Antihypertensive and vasorelaxant effects of FA

Maintaining hemodynamic parameters within physiological levels is essential for the proper functioning of the cardiovascular system [60]. In this context, BP, blood flow, and cardiovascular markers, including functionally relevant enzymes, constitute fundamental variables for the assessment of cardiac function [61]. Therefore, in the study by Salau et al. [12], the activity of enzymes such as AChE, ACE, and serum levels of creatine kinase myocardial band (CK-MB) were evaluated in vivo in rats with diabetic cardiomyopathy. As a result, they observed that treatment with FA (150 and 300 mg/kg, p.o. for 5 weeks) resulted in a decrease in the activity of AChE and ACE, in addition to reducing serum levels of CK-MB at both doses when compared with the group of animals with type II diabetes that received only distilled water, indicating that FA has therapeutic potential to attenuate the activity of AChE and ACE enzymes in experimental diabetes.

Similarly, FA was able to promote positive effects on cardiovascular parameters. In the study by Senaphan et al. [62], the authors investigated whether FA (for 6 weeks, 30 or 60 mg/kg/day, gavage) attenuated vascular inflammation and cardiovascular remodeling in rats fed a high-fat, high-carbohydrate diet. The results showed promising FA-mediated reductions in BP, OS, vascular inflammation, myocardial fibrosis, and cardiac hypertrophy mediated by Ang II. These effects were associated with reduced vascular superoxide production and lower plasma ACE levels, as well as suppression of type 1 receptor expression and vascular adhesion molecule 1, which correlate with improved hypertension and cardiac hypertrophy.

Therefore, FA has demonstrated a beneficial effect in regulating BP in diabetes. However, the specific role of FA in hypertension requires more experimental and clinical evidence, since, as demonstrated in the next topic, it exhibits robust antioxidant and anti-inflammatory capacities, but few studies have focused on the compound’s antihypertensive activity. Therefore, inferences about the function of FA in signaling pathways that mediate responses in the cardiovascular system have not yet been fully clarified.

### Anti-inflammatory and antioxidant effects of ferulic acid

In an in vivo study, Sprague Dawley rats underwent renal laparotomy surgery to induce ischemia/reperfusion kidney injury to evaluate the effect of FA treatment on inflammation and OS [63]. The groups were subdivided into: 4 h, 24 h, 48 h, and 7 days post-ischemia. Treatment was administered via FA (100 mg/kg p.o., 24 h before ischemia) or zinc oxide nanoparticles due to their anti-inflammatory and antioxidant effects (5 mg/kg i.p., 2 h before ischemia), or in a combined form (FA 100 mg/kg p.o., 24 h before ischemia and nanoparticle 5 mg/kg i.p., 2 h before ischemia) [63]. As a result, all three interventions were effective in reducing MDA concentrations at all time points analyzed, with a more pronounced and synergistic effect observed in the combined form [63]. Regarding catalase (CAT) concentrations, increases were observed at 24 h, 48 h, and 7 d, with the greatest effect in the combined intervention group. Concerning SOD concentrations, FA increased this enzyme only at 48 h and 7 d post-ischemia, while zinc nanoparticles and the dual intervention increased SOD levels at all time points [63].

In addition to ROS markers, the expression of antioxidant proteins HO-1 and NRF2 was also evaluated. The results show that both HO-1 and NRF2 were increased in all interventions, indicating activation of these important antioxidant pathways [63]. The same study also evaluated the expression of pro-inflammatory (TNF-α) and anti-inflammatory cytokines such as Hypoxia-Inducible Factor 1α (HIF-1α). A decrease in TNF-α was observed in all interventions, with FA showing better results compared to the nanocapsule group alone, and the synergism between them being evident at all time points analyzed [63]. In contrast, an immunohistochemical assay for HIF-1α messenger RNA (mRNA) expression revealed an increase in this protein in all interventions studied. Similar to the previous result, FA performed better than the nanocapsule group alone, and the combined treatment was the most effective at all time points analyzed [63].

Furthermore, Zhang et al. [13] evaluated the molecular mechanisms, particularly those related to oxidant activity, by which FA exerts the previously described cardioprotection in an experimental model of isoproterenol-induced heart failure. Plasma levels of OS markers [SOD, MDA, glutathione peroxidase (GSH-Px), and LDH release] were measured in male Sprague-Dawley rats subjected to treatment with different doses of FA (5, 25, and 50 mg/kg). Administration of FA resulted in an increase in the antioxidant enzymes SOD and GSH-Px, as well as a reduction in MDA and LDH levels, demonstrating a protective effect against OS. Notably, these effects were only observed at doses of 25 and 50 mg/kg in a dose-dependent manner [13].

In the same study, the authors also investigated the effect of FA on cardiomyocyte apoptosis. Through Western blot and TUNEL assay analysis, they observed that FA did not induce apoptosis or increase the expression of cleaved caspase 3/9 proteins in healthy animals. In contrast, FA administration markedly reduced apoptosis and cleaved caspase 3/9 expression in cardiomyocytes from animals with heart failure, indicating that FA attenuated cardiomyocyte injury [13].

Finally, after discovering that FA at a dose of 50 mg/kg activates the NRF2 pathway through increased p-NRF2, HO-1, and NQO1 and decreased Keap1, Zhang et al. [13] investigated whether FA under the same conditions could reduce SOD and MDA levels. Using the NRF2 pathway inhibitor ML385, it was observed that FA increased SOD concentrations and decreased MDA when administered alone, compared with or without the presence of ML385, relative to the heart failure + ML385 group. These results indicate that the antioxidant action of FA occurs, in part, through the NRF2 pathway.

In the context of non-alcoholic fatty liver disease, FA was further tested to evaluate its possible antioxidant activity [64]. Sprague-Dawley rats were fed a high-fat diet for 12 weeks for disease induction. Concurrently, FA treatment (20 mg/kg/day, p.o., for the last 4 weeks) was administered, after which serum and liver tissue samples were collected. Liver tissue was analyzed for SOD and MDA concentrations [64]. Using fluorescence microscopy, increased ROS levels (% area) were observed in the group receiving only the high-fat diet. However, in the group receiving the same diet combined with FA, reductions in ROS (%) and MDA were observed in both liver tissue (U/mg protein) and serum (U/mL). SOD levels, in turn, were increased in both conditions, further demonstrating the antioxidant activity of this compound [64].

From another perspective, given the anti-inflammatory capacity demonstrated by FA in different contexts, Wei et al. [64] analyzed FA and its impact on inflammatory biomarkers (IL-1β, IL-6, and TNF-α) in liver tissue in the experimental model described above. Using ELISA assays, it was reported that the concentrations of all analyzed cytokines were elevated in the group receiving only the high-fat diet, whereas the group receiving the diet in combination with FA (20 mg/kg/day, p.o., during the last 4 weeks of the diet) showed reduced levels of these markers, indicating a strong anti-inflammatory effect.

The study by Salau et al. [12], in turn, evaluated in vivo the protective effect of FA on diabetic cardiomyopathy in a Sprague-Dawley rat model of type 2 diabetes induced by 10% fructose and streptozotocin injection. Using a cardiac homogenate, biomarkers of OS, including GSH, CAT, and SOD, as well as MDA levels, were measured following FA treatment (150 and 300 mg/kg, orally, for 5 weeks). Treatment at the described doses effectively restored cardiac concentrations of GSH, CAT, and SOD and reduced MDA levels. Furthermore, FA improved the oxidative profile to near-normal values [12].

Subsequently, an in vitro study [65] evaluated the potential bioactivity of FA in inflammation and insulin resistance, as well as its mechanism of action in 3T3-L1 adipocytes treated with TNF-α. After inducing inflammation with TNF-α (50 ng/mL), there was a significant increase in three pro-inflammatory molecules: IL-6, IL-1β, and MCP-1. FA treatment resulted in a significant dose-dependent reduction in these markers [65]. Regarding inflammatory/anti-inflammatory activity, ELISA assays were used to measure the inhibitory effect of TNF-α release in RAW 264.7 macrophages stimulated with LPS and treated with FA (1, 10, and 50 µM for 24 h post-LPS). FA reduced TNF-α production in a dose-dependent manner [65].

In a controlled environment, Gomes Schmitt et al. [66] evaluated the bioactivity of Citrus sinensis extract (composition: FA, citric acid, and quercetin), orlistat, and their combination on biochemical (inflammatory and antioxidant) and hormonal parameters in obese Wistar females (induced by a high-fat diet for 9 weeks). Initially, ABTS and DPPH assays were performed to measure the extract’s radical-scavenging capacity. The extract at a concentration of 0.25 mg/mL achieved 58.79% ABTS radical inhibition (IC50). In contrast, in the DPPH assay (IC50), a concentration of 0.50 mg/mL resulted in 47.75% inhibition.

Furthermore, in vivo experiments evaluated obese rats treated with orlistat (1.72 mg/kg, p.o.), Citrus sinensis extract (7.15 mg/kg, p.o.), or a combination of both. Analysis of OS markers (GPx, CAT, and SOD) revealed that obese rats showed reduced levels compared to the eutrophic control. Treatment with the extract was more effective than orlistat alone in improving these three markers, with the extract alone increasing GPx and SOD to values higher than those of the eutrophic control [66]. Regarding markers of oxidative damage, TBARS and protein carbonylation, the best results were observed with the extract alone, followed by the combination therapy [66].

To evaluate anti-inflammatory capacity, inflammatory markers were measured across the interventions. Both orlistat and the extract were able to reduce IL-1β, IL-8, IL-6, and TNF-α levels, and increase IL-10, with the extract demonstrating more pronounced effects [66].

In another experimental condition, diabetic nephropathy was analyzed to verify the anti-inflammatory bioactivity of FA in vivo [67]. Diabetic nephropathy was induced in male C57BL/6J mice by a high-fat diet combined with streptozotocin injection (35 mg/kg, i.p., for 5 consecutive days). Mice were treated with FA (200 mg/kg, p.o., for 8 weeks), and valsartan (12 mg/kg) was used as a positive control. Immunohistochemical assays demonstrated that both FA and valsartan significantly reduced NLRP3 and IL-1β expression compared to the diseased control group [67], indicating anti-inflammatory effects. Western blot analysis reinforced these findings, confirming that both interventions inhibited the expression of these proteins and, consequently, reduced inflammation.

Therefore, based on evidence from different experimental pathologies, FA appears to be a promising therapeutic compound capable of attenuating OS and inflammation in cardiovascular and metabolic diseases. However, the limited number of studies in humans prevents definitive conclusions regarding whether these responses will also occur in patients.

### Toxicity of CA and its derivatives

CA and its derivatives, both in isolated form and in extracts, have demonstrated favorable safety profiles in preclinical models, showing well-tolerated therapeutic doses and effective antioxidant compensation mechanisms. In C57BL/6J mice, CA administered at 300 mg/kg/day orally for 14 days did not induce toxicity [45]. Similarly, CA nanoparticles dissolved in olive oil (60 mg/kg, orally) were shown to be safe in pancreatitis models [47].

The CytoTox-Glo cell membrane integrity assay was employed to assess the hepatotoxic potential of the CA derivatives (R, S)-(E)-N-(1-hydroxy-3-methylbutan-2-yl)cinnamamide, (E)-3-(4-chlorophenyl)-N-(1-hydroxy-2-methylpropan-2-yl)acrylamide, and (E)-1-(4-hydroxypiperidin-1-yl)-3-phenylprop-2-en-1-one) [52]. Human HepG2 hepatocellular carcinoma cells were incubated with these compounds (1–50 µM) for 24 h. The results demonstrated that the derivatives did not compromise cell membrane integrity, and were considered non-hepatotoxic, non-cardiotoxic, and non-mutagenic [52].

Iftikhar et al. [51] further evaluated oral toxicity of Citrullus colocynthis fractions containing CA derivatives. Doses of up to 2500 mg/kg (p.o.) were administered, and animals were monitored for 14 days. No behavioral changes, toxicity, or deaths were observed, confirming the safety of the extract [51].

Zielinska et al. [35] assessed CAF (50 µM) in colon myofibroblasts stimulated with IL-1β. Cell viability assays indicated 90% viability compared to the control, suggesting the absence of cytotoxicity at the tested concentration [35]. Likewise, FA was tested in 3T3-L1 adipocytes using an MTT assay. FA at 1, 10, and 50 µM did not affect cell viability, confirming non-cytotoxicity at these doses [65].

Collectively, these studies indicate that CA and its derivatives exhibit a favorable safety profile. Nevertheless, additional experimental studies in animals and humans, covering a broader range of doses, are required to fully confirm the safety of these compounds.

### Bioavailability of CA, CAF, and FA

CA, CAF, and FA are not classified as pharmaceutical drugs; therefore, there is no standardized dosage regimen or established dose definition for their clinical application or use in experimental models, which compromises their proper biopharmaceutical classification [68]. In this context, considering that bioavailability corresponds to the fraction of the bioactive compound or nutrient effectively absorbed in the gastrointestinal tract and made available in the systemic circulation [69], this parameter plays a central role in determining the effective dose and maximizing the expected biological response.

CAF and FA have low molecular weight and a relatively nonpolar character, especially in their non-ionized forms, which favors passive diffusion through the gastric mucosa [70], enabling their absorption and partial degradation in the stomach [71]. Studies demonstrate that plasma concentrations of FA in the portal vein peak approximately 5 min after oral administration, indicating rapid absorption in both the stomach and small intestine [72]. The absorption of CAF and FA occurs predominantly by passive diffusion and may also involve transporter-mediated mechanisms [68]. CAF exhibits high intestinal bioavailability, with up to 95% of its free form being absorbed by the intestinal mucosa [73]. CA is absorbed mainly in the intestine, with ion transport mediated by enterocytes of the ileum and jejunum, as demonstrated in murine models [74]. Additionally, Veras et al. [68] observed that the absorption rate of CA is higher than that of FA.

The absorption and distribution of phenolic compounds depend on their metabolism, which occurs in the gastrointestinal tract, liver, and kidneys. Due to the rapid and extensive metabolism of CA and its derivatives, most metabolites are rapidly excreted in the bile and urine, and in smaller quantities in the feces [72].

Regarding the bioavailability of CA and its derivatives in humans, studies evaluating the total plasma concentration of phenolics over time (µmol/L·h) have demonstrated variations according to the food matrix and the administered dose. Consumption of rosemary tea (1.2–1.8 mg/kg body weight) resulted in a total plasma concentration of CAF of 2.85 µmol/L·h [75], whereas ingestion of common beans reached 15.4 µmol/L·h [76]. For FA, porridge enriched with rice bran (0.346 mg/kg body weight) showed a total plasma concentration of 0.8299 µmol/L·h [77], and ingestion of mango pulp (*Mangifera indica* L.) resulted in 0.0077 µmol/L·h [78]. Regarding CA, ingestion of 5 g of *Cinnamomum zeylanicum* resulted in a plasma concentration of 588 µmol·min/L [79]. In general, the bioavailability of CAF, FA, and CA varies according to the food source, dose, and matrix, highlighting the influence of factors such as digestion and metabolism on the plasma kinetics of these compounds. Thus, methodological standardization is essential to better understand their biological relevance and therapeutic potential in humans.

Thus, the effects of CA, CAF, and FA on AH and its pathophysiological outcomes depend critically on their plasma concentrations and distribution in tissue compartments. In this sense, it is essential to conduct comprehensive preclinical and clinical pharmacokinetic studies encompassing the processes of absorption, distribution, metabolism, and excretion in order to robustly establish therapeutically effective doses of these acids and to estimate their oral bioavailability more precisely, a parameter that remains insufficiently elucidated.

## Conclusion

**Fig. 3:**
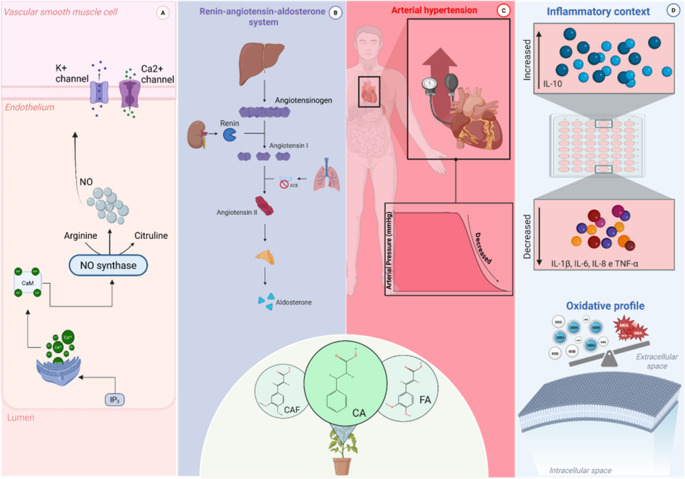
Graphical abstract. Cinnamic, caffeic, and ferulic acids protect the cardiovascular system by enhancing endothelial nitric oxide signaling, inhibiting ACE, reducing pro-inflammatory cytokines, and activating antioxidant defenses, collectively improving vascular function and lowering blood pressure

Integrative experimental evidence indicates that the cardioprotective effects of CA and its derivatives arise from their ability to modulate three interconnected physiological systems: (1) the vascular–endothelial axis, through regulation of nitric oxide (NO) and angiotensin-converting enzyme (ACE); (2) the oxidative–inflammatory interface, via activation of NRF2/HO-1 and suppression of NF-κB; and (3) the metabolic–mitochondrial axis, by improving mitochondrial membrane potential and reducing superoxide generation. This triad underlies the consistent improvements in endothelial function, cardiac remodeling, and vascular homeostasis observed across various experimental models (summarized in Fig. 3).

Thus, CA and FA emerge as promising natural compounds capable of modulating multiple physiological pathways involved in blood pressure regulation, while CAF appears to exert more modest or indirect effects on BP. Their multifaceted actions—including antihypertensive, antioxidant, anti-inflammatory, and endothelial-protective effects—highlight their potential as candidates for the development of novel therapeutic strategies aimed at mitigating hypertension and its cardiovascular complications. It is important to emphasize that these conclusions are drawn from preclinical studies, and whether these responses translate to humans remains to be determined.

### Recommendations for future research

Given the promising preclinical safety and efficacy profiles of cinnamic acid (CA) and ferulic acid (FA), these compounds represent strong candidates for development as adjunctive therapies or molecular scaffolds for novel antihypertensive and metabolic drugs. In contrast, caffeic acid (CAF) appears to exert limited or indirect effects on blood pressure regulation and may be less relevant as a primary therapeutic agent for hypertension.

To facilitate clinical translation, future research should focus on:

1. Isolated Compound Studies: Evaluating CA and FA in their pure forms to accurately determine their pharmacological effects without confounding influences from extract matrices.
2. Pharmacokinetics and Bioavailability: Assessing absorption, distribution, metabolism, and excretion to optimize dosing strategies.
3. Dose-Response and Safety Profiling: Establishing effective and safe therapeutic windows in relevant animal models.
4. Synergistic Potential: Investigating potential interactions with existing antihypertensive medications to explore combination therapies.
5. Mechanistic Clarification: Further elucidating molecular pathways, particularly in oxidative stress, inflammation, and endothelial function, to identify precise targets for intervention.

By addressing these areas, future studies can bridge the gap between preclinical findings and clinical application, providing a foundation for the development of safe and effective phenolic acid-based therapies for hypertension and related cardiovascular disorders.

## Data Availability

No datasets were generated or analysed during the current study.

## Abbreviations

ABTS: 3-ethylbenzothiazoline-6-sulfonic acid
ACE: angiotensin-converting enzyme
AChE: acetylcholinesterase
AH: Arterial hypertension
Akt: protein kinase B
AMPK: AMP-activated protein kinase
Ang II: Angiotensin II
ASK1: Apoptosis Signal-Regulating Kinase
BP: blood pressure
BuChE: butyrylcholinesterase
CA: cinnamic acid
Ca^2+^: calcium ion
CAF: caffeic acid
CaM: calmodulin
CAT: catalase
CK-MB: creatine kinase myocardial band
DBP: diastolic blood pressure
DPPH: 2,2-diphenyl-1-picrylhydrazyl
DryAr: extract obtained from dried fruits
eNOS: endothelial nitric oxide synthase
ERK 1/2: extracellular signal-regulated kinases 1/2
FA: ferulic acid
FrozAr: obtained from frozen fruits
GLUC: High glucose
GSH: Reduced glutathione
GSH-Px: glutathione peroxidase
GSSG: Oxidized glutathione
H₂O₂: hydrogen peroxide
HIF-1α: Factor 1 Hypoxia-inducibleα
HO-1: heme oxygenase-1
IL-10: Interleukin 10
IL-1β: Interleukin 1 beta
IL-6: Interleukin 6
IL-8: Interleukin 8
IP3: inositol triphosphate
JNK: c-Jun N-terminal kinase
K^+^: potassium ion
LDH: lactate dehydrogenase
LPS: lipopolysaccharide
MAP: Mean Arterial Pressure
MAPK: mitogen-activated protein kinase
MDA: malondialdehyde
MMP: Mitochondrial membrane potential
MPO: myeloperoxidase
mRNA: Ribonucleic Acid messenger
MTT: 3-(4,5-dimethylthiazol-2-yl)-2,5-diphenyl tetrazolium bromide
NE: norepinephrine
NF-κB: Nuclear factor kappa B
NLRP3: NLR family pyrin domain containing 3
NO: nitric oxide
Nppb: Natriuretic Peptide Type B
NPSH: Non-Protein Sulfhydryl Groups
NQO1: (NAD(P)H Quinone Dehydrogenase 1)
Nrf2: nuclear factor erythroid 2
O^2−^: Oxygen Ion
OS: oxidative stress
p.o: oral route
PGI2: prostaglandin I2
PPARs: peroxisome proliferator-activated receptors
PPARs: peroxisome proliferator-stimulating agents
PPARδ: Peroxisome Proliferator-Activated Receptor delta
RAAS: Renin-angiotensin-aldosterone system
ROS: reactive oxygen species
ROS: reactive oxygen species
SBP: systolic blood pressure
SHR: Spontaneously hypertensive rat
SOD: superoxide dismutase
STAT3: transducer Signal and transcription activator 3
TAC: total antioxidant capacity
TBARS: thiobarbituric acid reactive species
TNF-α: Tumor Necrosis Factor alpha
T-SH: Total Sulfhydryl Groups
